# Pressure Measurements Obtained from Intraosseous Access: Potential Clinical Applications Explored Using a Porcine Model

**DOI:** 10.1007/s12265-025-10719-7

**Published:** 2026-02-06

**Authors:** Joerg Reifart, Nida Asif, Paul Iaizzo

**Affiliations:** 1 Visible Heart® Laboratories, Department of Surgery, Institute for Engineering in Medicine, University of Minnesota, B172 Mayo, MMC195 | 420 Delaware Street S.E., Minneapolis, MN 55455 USA; 2 Cardiovascular Department, M Health Fairview University of Minnesota Medical Center East Bank, Minneapolis, MN USA; 3 University of the Punjab, Punjab, Pakistan

**Keywords:** Intraosseous pressure, Medullary pressure, Arterial pressure, Cardiac arrest, Intraosseous access, CPR, PEA, Pressure prediction

## Abstract

Intraosseous access, the fastest access in emergencies, is exclusively used for delivering medications or fluids. The correlation between intraosseous and arterial pressures remains unclear. This study aimed to explore this correlation at baseline and in various clinical scenarios (e.g., different heart rates, arrhythmias, asystolic arrest, and CPR). In 11 male Yorkshire pigs (73.4 ± 5.9 kg), femoral artery and tibial Intraosseous lines were placed under anesthesia. Pressures were recorded during hemodynamic interventions and cardiac arrest. Analyses included Pearson’s r, Wilcoxon rank-sum test, and BVAR. Intraosseous pressure showed correlating pulsatility with arterial pressure, ranging from 9 to 71% of mean arterial pressure. Correlation was strong under normal conditions (r = 0.75–0.96, p < 0.001) and during CPR (r = 0.65–0.99, *p* < 0.001), weakened during asystole (r = 0.26 ± 0.46, *p* < 0.001), and was disrupted by epinephrine (r = 0.04, *p* < 0.001). Asystole was identifiable on intraosseous tracings. Intraosseous pressure effectively reflects circulatory activity and may aid in accurately identifying asystole with possible clinical implications for CPR.

**Graphical Abstract:**

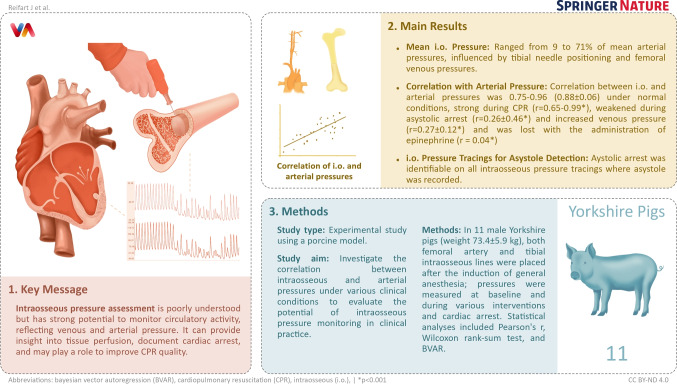

**Supplementary Information:**

The online version contains supplementary material available at 10.1007/s12265-025-10719-7.

## Introduction

In critical care, continuous waveform monitoring of arterial blood pressure (intraarterial blood pressure) is the most relied-upon surrogate for the perfusion of vital organs and circulatory activity [1]. Even in ideal settings, intraarterial access, cannulation, and set-up take several minutes and can have significant failure rates. Further, arterial access is not frequently performed in prehospital settings. In contrast, intraosseous access is the fastest access to the vascular system and is known to have a lower failure rate in emergent situations compared to venous access [2–4]. In several guidelines, intraosseous access use is suggested as a reasonable alternative to intravenous access in cardiopulmonary resuscitation or advanced trauma life support [5–7].

Though intraosseous access is clinically established and can be easily deployed in prehospital settings, pressure measurements derived from this access are not used. Currently, there is a paucity of knowledge regarding the interpretation and clinical application of measured intraosseous pressure though some prior studies have evaluated the use of IO access to monitor blood pressure [8, 9].

In the present study, we sought to explore the potential value of obtaining pressure readings from intraosseous access by investigating whether intraosseous pressures correlate with arterial pressures in different clinical scenarios and what factors may contribute to differing pressures.

## Methods

### Study Ethics

This research was conducted as part of a protocol that was approved by the Institutional Animal Care and Use Committee of the University of Minnesota and was compliant with the National Research Council's 2011 Guidelines for the Care and Use of Laboratory Animals (Protocol No. 2305-41090A). Clinical trial number: not applicable.

### Study Preparation

This study was an exploratory pilot with no prior data to guide a formal sample size calculation. We selected 11 animals based on alignment with similar hemodynamic studies. Healthy male Yorkshire swine (73.4 ± 5.9 kg) with an average age of 6 months that had previously acclimated to the animal facility setting for 2 −6 weeks and fasted for 12 h, were sedated with Telazol (≤ 500 mg/kg) intramuscularly, followed by Methohexital (≤ 50 mg/kg) after establishing intravenous access. After intubation, anesthesia was maintained with a 1‐to‐1.5% minimum alveolar concentration (MAC) of isoflurane during continuous ventilation. All animals received periodic intravenous boluses of 5,000 IU of Heparin with a total administration of 30,000 IU.

**Fig. 1:**
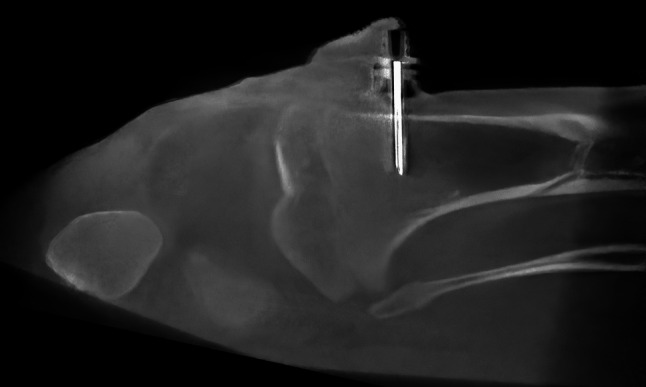
Micro-computed tomography reconstruction of a 25 mm intraosseous access within the proximal porcine tibia of one of the animals

In each animal, the left femoral artery was cannulated with an 18 G 1–3/4″ Jelco IV catheter (Smiths Medical, Minneapolis, MN, USA) for continuous intraarterial pressure (IAP) monitoring. For venous access, a 12.5 French, 13 cm long SafeSheath® II Hemostatic Peel-away Introducer System for Vascular Access (Medtronic, Minneapolis, USA) was used in the right femoral vein and right internal jugular vein. The position of the catheter in the inferior vena cava was confirmed with fluoroscopy. Intraosseous access was gained using a 25 mm,15G EZ-IO® device (Teleflex®, Inc., PA, U.S.A.), drilling it into the proximal tibia of the same side as the femoral arterial access, unless the intraosseous access was not successful (Fig. 1). The lines were flushed with 10 ml heparinized saline (10 IU/ml); no continuous infusions were attached to the lines.

Intraosseous cannulation was deemed successful when there was a palpable loss of resistance, the needle remained upright, blood could be withdrawn, and saline flush was possible [10]. A successful intraosseous pressure reading was defined as a pulsatile pressure signal under resting conditions.

All measurements were done with the animals in the supine position. The intraosseous, intraarterial, and intravenous lines (in case of central venous pressure) were zeroed at the approximate mid-axillary level. Pressure calibration was calibrated at 0 and 200 mmHg using a Utah Medical Delta-Cal 650–950 (Utah-Medical West Midvale, Utah, USA) transducer simulator and tester.

Simultaneous data were recorded using a physiological data monitoring system (EMKA Technologies, Paris, France), with data points continuously recorded every 2 ms. These pressure measurements underwent denoising with a 10 Hz filter.

### Comparative Analyses of the Relationships between Arterial and Intraosseous Pressures

To describe and test the temporal and relative pressure relationships between recorded intraarterial and intraosseous pressures, continuous recordings were shortened to 40-s measurements. Before analyzing a given data set, time differences in pulse peaks (phase shift due to different lengths of the fluid-filled line to the transducer) were corrected (Temporal phase correction by 30–60 ms).

Arterial pressures were compared to intraosseous pressure at baseline (normotension *n* = 6), during: 1) anesthesia-induced hypotension induced by (systolic blood pressure < 90 mmHg occurring during prolonged anesthesia, *n* = 2), 2) right jugular transvenous ventricular cardiac pacing at rates of 120/min (right ventricular screw-in lead via the right jugular vein *n* = 1), 130 (*n* = 1), 140 (*n* = 2, Supplementary Fig. 1), 3) pacing-induced ventricular arrhythmias (*n* = 1, Supplementary Fig. 2), 4) ventilator-induced breath hold (breath hold was performed by locking the ventilator in an inspiratory hold for up to 30 s *n* = 2); 5) temporary balloon occlusion of the distal inferior vena cava to increase femoral vein pressures (IVC occlusion with a TORAY PTMC-30 Inoue-Balloon fluoroscopically positioned, *n* = 1, Supplementary Fig. 3), and 6) administration of an epinephrine bolus during spontaneous circulation with stable hemodynamics (200 µg i.v., *n* = 1).

### Intraosseous Pressure Changes Associated with Loss of Spontaneous Circulation

All studies ended in asystole, confirmed by loss of intraarterial pressures. Simultaneous intraarterial and intraosseous pressure recordings were continued during the initiation of cardiac arrest via infusion of St Thomas’ Hospital cardioplegic solution in 6 cases [11]. This led to reproducible patterns of normotension transitioning to hypotension with values < 60 mmHg, analogous to what is clinically seen in pseudo-PEA with the final transition to complete pulselessness and asystole (Figure 4).

Finally, one case of cardiac arrest with subsequently administered low-quality cardiopulmonary resuscitation (CPR) with an automatic piston chest compression device programmed at 110 compressions per minute and a depth of 5 cm, resulting in sustained arterial diastolic pressure < 25 mmHg, was recorded [12].

### Statistical Analyses

All pressure values were presented as means ± standard deviations. Statistical analyses were done with R version 3.6.1. Bayesian vector autoregression estimates were calculated using EViews 13.

We calculated Pearson's correlation coefficients for arterial and intraosseous pressure and modeled the dynamic links between intraosseous and intraarterial pressures using Bayesian VAR estimations.

To evaluate abilities to detect cardiac arrest on both arterial and intraosseous pressure tracings, peak pulse pressures (pmax – pmin) before and after arrest were calculated and compared using Wilcoxon rank-sum tests.

A *p*-value of less than 0.05 was considered statistically significant.

A post-hoc power analysis based on our observed baseline correlation (r ≈ 0.80) confirmed that the sample size was sufficient to detect a strong correlation with 80% power. Subgroup analyses remain exploratory, given the sample size.

## Results

Intraosseous access was successful in all 11 attempted cases. The needle was reinserted in two cases, as there were no pulsatile pressure tracings in the initial attempts.

Mean intraosseous pressure ranged from 5 to 36 mmHg, reflecting 9 to 71% of the measured mean arterial pressures.

### Correlations between Arterial and Intraosseous Pressures

Intraosseous and intraarterial pressures correlated strongly in all described non-pharmacologic scenarios with stable venous pressures; the correlation coefficients in these scenarios ranged from 0.66 to 0.99 (see Table 1). Similarly, systolic and diastolic pressures showed a strong positive correlation (0.52–0.97), unless epinephrine was administered (Supplementary Table 1). When we induced increased venous pressure via balloon occlusion of the distal inferior vena cava, diastolic intraosseous pressure rose linearly by 17 mmHg, while pulse pressure remained unchanged (17.4 mmHg before and 17.5 mmHg after inflation), owing to a similar increase in both systolic and diastolic pressures (Supplementary Fig. 3). Before IVC occlusion, there was a strong correlation between arterial and intraosseous pressure (r = 0.91, *p* < 0.005), which lessened during IVC pressure increase (r = 0.33, *p* < 0.005) and then re-increased after pressures stabilized (r = 0.82, *p* < 0.005).

**Table 1:** Pearson’s correlation coefficient and Bayesian VAR estimation results of different clinical situations

Clinical scenario	Mean I.A. pressure (mmHg)	Mean I.O. pressure (mmHg)	Pearson’s r	R-squared	Adj. R-squared	Sum sq. resids	S.E. equation	F-statistic
Normotension (*n* = 6)	67.5 ± 13.7	34.3 ± 5.4	0.9	0.996163	0.996163	233.8144	0.111299	2450284
Hypotension (*n* = 2)	54.6 ± 13.9	25.7 ± 2.7	0.98	0.997733	0.997733	475.6022	0.128409	6346379
Pacing 140 bpm (*n* = 2)	64.9 ± 15.2	28.3 ± 2.9	0.97	0.996954	0.996954	267.4006	0.121369	2970724
Ventricular tachycardia (*n* = 1)	66.0 ± 15.7	38.4 ± 3.8	0.92	0.998825	0.998825	181.7435	0.129609	4597984
CPR (*n* = 1)	29.8 ± 7.5	54.9 ± 0.6	0.6	0.963543	0.963542	739.3324	0.080631	1001850
Breath hold (*n* = 1)	83.4 ± 12.0	38.4 ± 3.8	0.85	0.981097	0.981091	308.7534	0.223175	1608734
Epinephrine (200 mg)(*n* = 1)	97.7 ± 29.8	30.9 ± 4.5	0.03	0.997714	0.997714	906.2173	0.214745	4288540

**Fig. 2:**
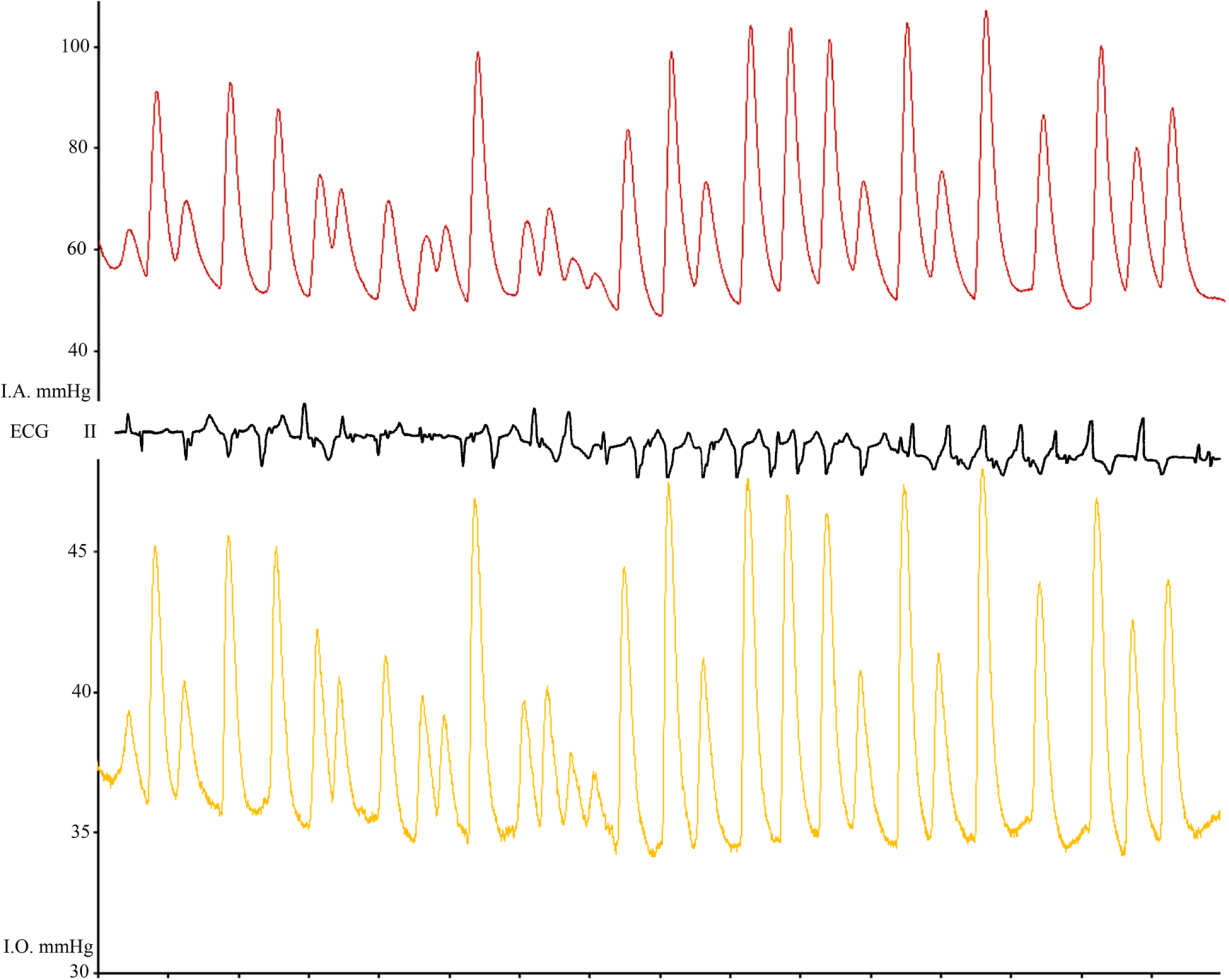
Pressure and ECG tracings of one pig in which ventricular tachycardia was induced. The tracings show a strong correlation (Pearson’s r = 0.96, *p* < 0.05)

**Fig. 3:**
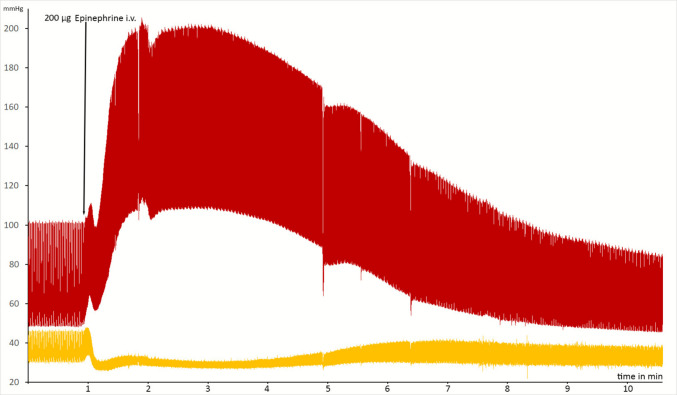
Recording of intraosseous and arterial pressure before and after administration of epinephrine in one animal. As arterial pressure increased, intraosseous pressure dropped

An induced ventricular arrhythmia did not result in diminished correlations (see Fig. 2). Yet, the administration of epinephrine led to reduced correlation upon onset of the pharmacologic responses, with recovery after 6- 10 min (see Fig. 3). High correlation coefficients were obtained in cases with low or high baseline intraosseous pressure values. Results of Bayesian VAR estimates suggested strong correlations in all tested clinical scenarios (see Table 1). All calculated correlation coefficients were statistically significant at a *p* < 0.001.

### Intraosseous Pressures during the Loss of Spontaneous Circulation

**Fig. 4:**
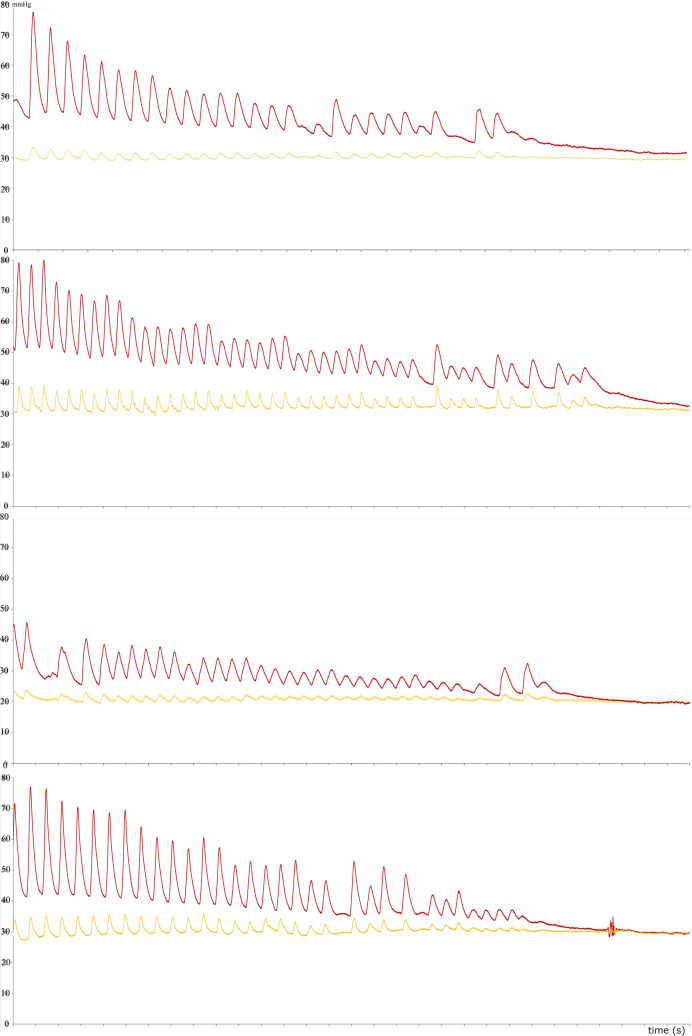
Intraosseous and arterial pressure tracing during the induced loss of spontaneous circulation in four different animals

Loss of pulsatility associated with asystolic cardiac arrest was distinguishable in all animals (see Fig. 4). Yet, intraosseous and arterial pressure correlations upon cardiac arrest varied substantially (−0.56 to 0.82, mean r = 0.26 ± 0.46) and were overall less strong in this clinical scenario due to differences in mean pressure trajectories (pressure differences equalizing during asystole).

The last cardiac contraction that generated a deviation in the pressure tracing before asystole occurred at a mean systolic peak of 35.6 ± 6.7 mmHg in the intraarterial and 29.5 ± 6 on the intraosseous line. Mean peak pulse-pressures before and after arrest were 15 ± 8.4 mmHg and 3.7 ± 1.1 mmHg (*p* = 0.005) for arterial, compared to 4.2 ± 2 mmHg and 1.2 ± 0.3 mmHg (*p* = 0.002) for intraosseous pressures.

All final cardiac induced pulses present on arterial pressure tracings were identifiable on the intraosseous pressure tracings.

### Intraosseous Pressure during CPR

Applied chest compression during CPR after cardiac arrest due to ventricular fibrillation led to maximum diastolic intraarterial pressures of 21 mmHg. Pulsatility was seen in both arterial and intraosseous pressure tracings. There was a positive linear correlation between intraosseous and intraarterial pressure, exceeding a correlation coefficient of 0.7 and reaching 0.99 at different times during administered CPR (r = 0.65–0.99, *p* < 0.001).

## Discussion

Intraosseous pressure (also referred to as intramedullary pressure), has been of interest in both orthopedic and hemoncologic research in the last 90 years [13–16].

Larsen was among the first to describe its synchronicity with carotid pressure and showed that intraosseous pressure decreases after epinephrine administration in a canine model [15]. More recently, Frascone et al. demonstrated parallel declines in intraosseous, arterial, and central venous pressures during progressive hemorrhagic hypotension in swine [17].

In humans, Salzman et al. and Frascone et al. reported simultaneous intraosseous and non-invasive arterial pressure measurements [8, 18]. Salzman’s study of 15 participants noted significant pressure drift—unlike earlier studies [14, 19].

Our study builds on this foundation, representing one of the first to correlate intraosseous with intraarterial pressure across varying physiological states. While intraosseous pressure does not directly predict arterial pressure, it shows potential as a surrogate for perfusion, especially in peri-arrest contexts. Three main factors limit its use as a direct predictor: (1) adrenergically mediated vascular resistance, (2) venous pressure shifts, and (3) needle positioning.

### Intraosseous Pressure during Loss of Spontaneous Circulation

Our findings suggest intraosseous pressure may supplement manual pulse checks in critical settings. Absence of pulsatility on the intraosseous pressure tracing may signal the need for CPR, potentially improving decision-making where manual checks—shown to be inaccurate in up to 14% of pediatric arrests—are unreliable [20]. Low but perfusing pressures may be missed, prompting unnecessary CPR and its risks, especially in prehospital settings where reliance on manual checks remains high [21].

We observed intraosseous pressure pulsatility at systolic pressures < 40 mmHg, consistent with pseudo-PEA, sometimes defined as SBP < 60 mmHg [22]. Intraosseous tracings may help distinguish pseudo-PEA from true PEA and asystole, guiding therapy appropriately [23].

These scenarios, particularly in the prehospital environment where intraosseousaccess is common, may benefit from incorporating intraosseoustracings in clinical decisions.

### Intraosseous Pressure during CPR

There remains a need for real-time hemodynamic monitoring during CPR [24]. Though intraarterial access provides insight into compression quality, its clinical use is limited by procedural time, training requirements, and bleeding risk [25, 26].

Given elevated venous pressures during CPR (25–30 mmHg) and sympathetic vasoconstriction, intraosseous pressure was thought unlikely to show pulsatility [27, 28]. Yet in our model, pulsatility was consistently observed and correlated with intraarterial pressure.

Notably, CPR quality was low (diastolic pressures < 25 mmHg), suggesting that intraosseouspressure tracings may still be informative, though further studies are needed to determine their utility in guiding resuscitation [12].

### Limitations

This study was conducted in a controlled porcine model with a small sample size, limiting generalizability. Despite some human data, clinical translation requires caution.

Baseline intraosseous pressure variability could reflect technical factors. Incorrect intraosseous placement occurs in up to 20% of adults and 11–64% of pediatric cases, depending on the device [29–33]. Drawing conclusions from an incorrectly placed intraosseous line could be detrimental to patients.

No medications or fluids were administered through the intraosseous line, and no epinephrine was given during CPR. Since epinephrine alters hemodynamic correlations, our CPR findings may not apply to epinephrine-treated scenarios.

Isoflurane anesthesia, known to influence peripheral vascular tone, could have affected intraosseous pressures [34, 35]. It is also unclear whether heparin is necessary to maintain line patency; Salzman et al. observed signal loss, possibly from clotting [8].

Lastly, standard statistical methods like Pearson’s r may not capture the relationship between time-series pressure waveforms, limiting interpretability.

## Conclusion

While intraosseous pressure measurement cannot replace arterial pressure monitoring, it correlates well with arterial pressure and may serve as a surrogate marker of circulatory activity—particularly for pulse detection. Further studies are needed to explore its role in critical care settings.

## Supplementary Information

Below is the link to the electronic supplementary material.

Supplementary file1 Ventricular pacing at 140 bpm in one animal, correlation: Pearson’s r = 0.84, *p*<0.05. Intraosseous pressure in yellow. Femoral arterial pressure in red (PNG 583 KB)

Supplementary file2 Ventricular arrythmia in one animal. Intraosseous pressure in yellow. Femoral arterial pressure in red (PNG 478 KB)

Supplementary file3 Intraosseous pressure during inflation of balloon to occlude inferior vena cava in one animal with resulting increase in venous pressure (CVP) and its effects on intraosseous pressure (yellow) and arterial pressure (red) (PNG 215 KB)

Supplementary file4 (DOCX 18 KB)

## Data Availability

All data can be made available upon request to the first author when submitted alongside an analysis proposal.

## Declarations

### Data in Brief

All data is available upon reasonable request.
