# Supplementary figures and images for: Pressure Measurements Obtained from Intraosseous Access: Potential Clinical Applications Explored Using a Porcine Model
Source: J Cardiovasc Transl Res. 2026 Feb 6;19(1):21. doi: 10.1007/s12265-025-10719-7 (PMC12881039; doi:10.1007/s12265-025-10719-7)

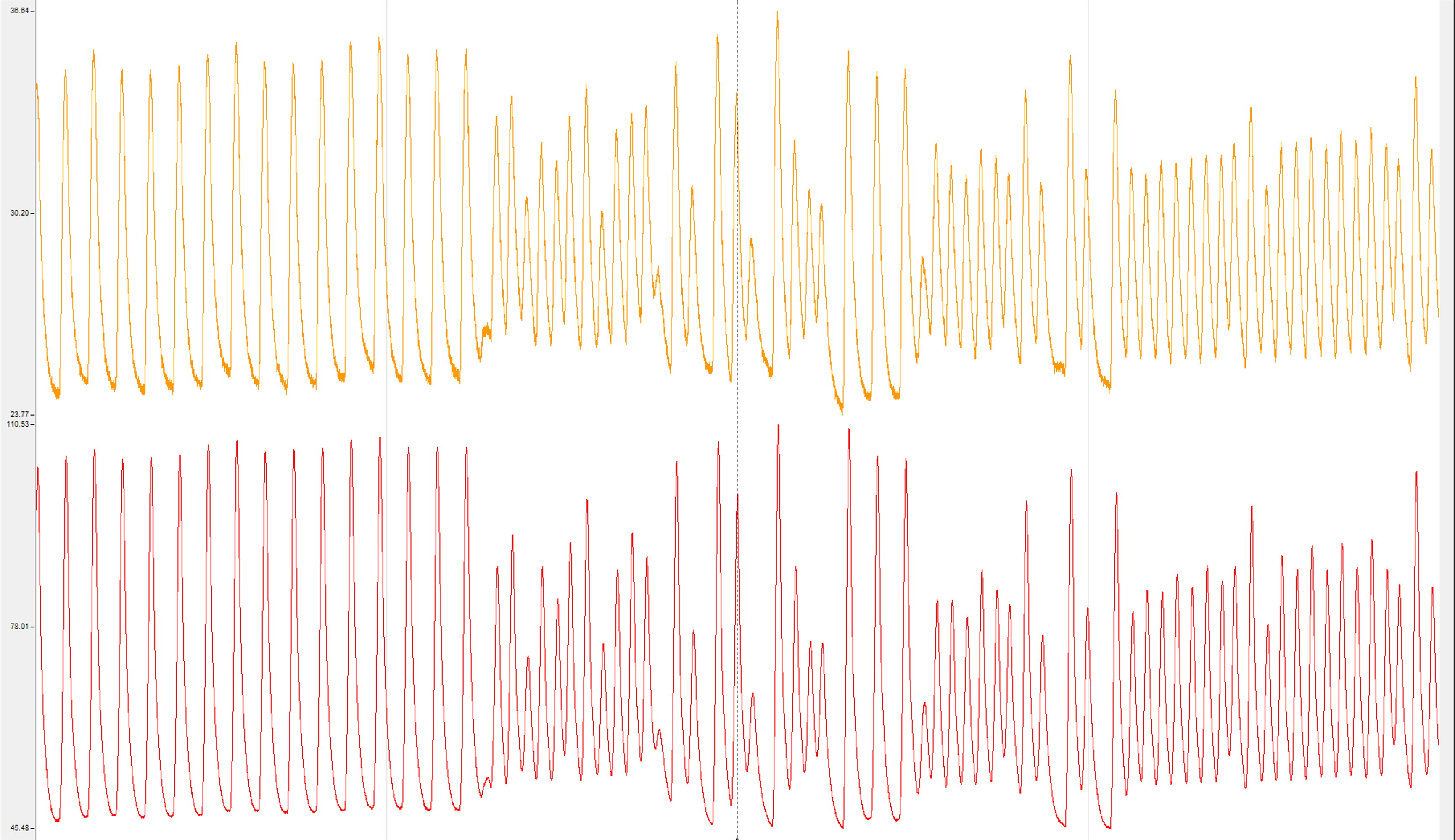

Supplement: Supplementary file 1 — Supplementary file1 Ventricular pacing at 140 bpm in one animal, correlation: Pearson’s r = 0.84, p<0.05. Intraosseous pressure in yellow. Femoral arterial pressure in red (PNG 583 KB) [file 12265_2025_10719_MOESM1_ESM.png]

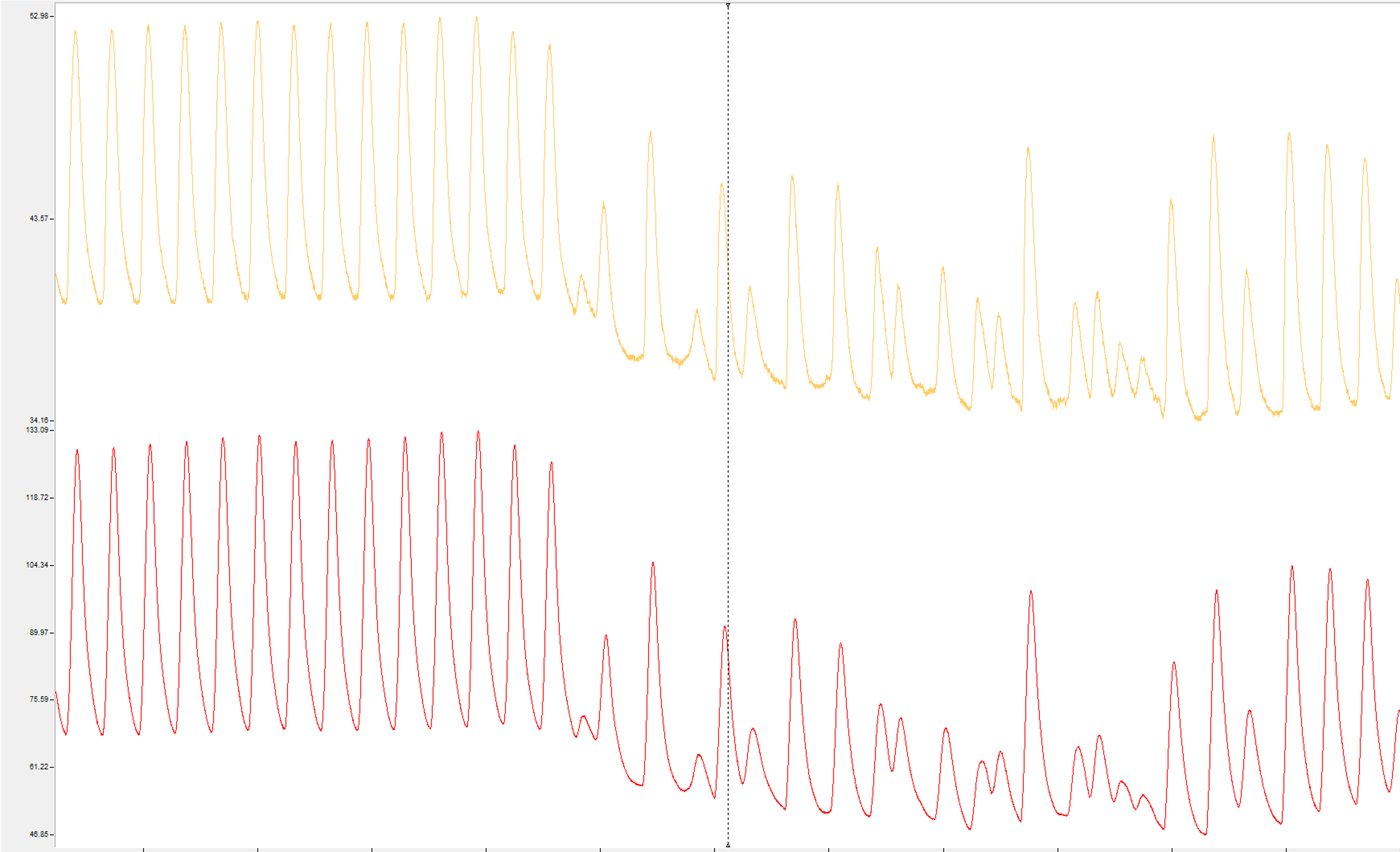

Supplement: Supplementary file 2 — Supplementary file2 Ventricular arrythmia in one animal. Intraosseous pressure in yellow. Femoral arterial pressure in red (PNG 478 KB) [file 12265_2025_10719_MOESM2_ESM.png]

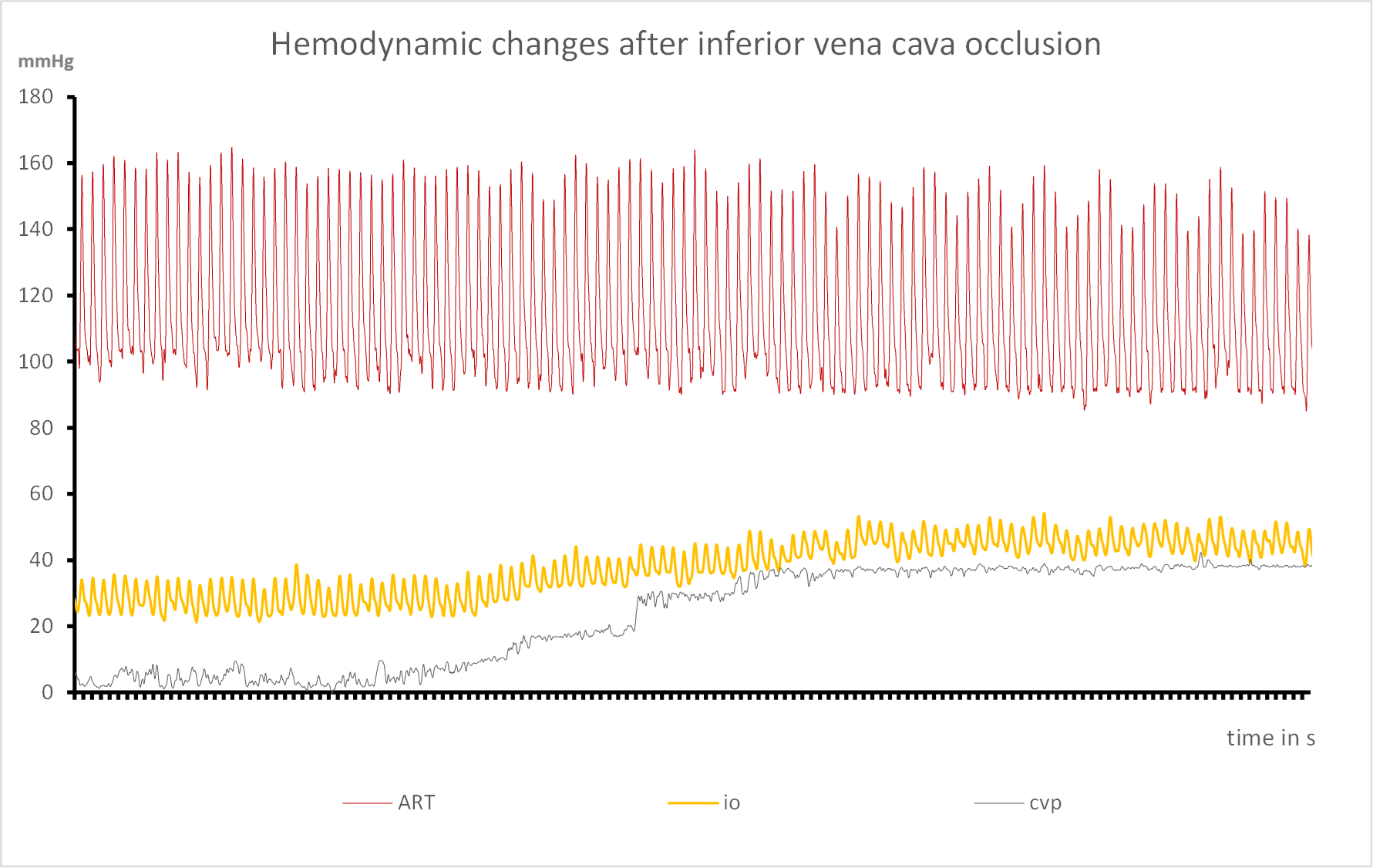

Supplement: Supplementary file 3 — Supplementary file3 Intraosseous pressure during inflation of balloon to occlude inferior vena cava in one animal with resulting increase in venous pressure (CVP) and its effects on intraosseous pressure (yellow) and arterial pressure (red) (PNG 215 KB) [file 12265_2025_10719_MOESM3_ESM.png]
