# Supplementary material for: Pressure Measurements Obtained from Intraosseous Access: Potential Clinical Applications Explored Using a Porcine Model
Source: J Cardiovasc Transl Res. 2026 Feb 6;19(1):21. doi: 10.1007/s12265-025-10719-7 (PMC12881039; doi:10.1007/s12265-025-10719-7)
Supplement: Supplementary file 4 — Supplementary file4 (DOCX 18 KB) [file 12265_2025_10719_MOESM4_ESM.docx]

**Table 1: Pearson’s correlation coefficient for arterial and intraosseous systolic and diastolic values**

| **Clinical scenario** | **Median (IQR) I.A. pressure (mmHg)** | **Median (IQR) I.O. pressure**  **(mmHg)** | **Mean systolic I.A. pressure (mmHg)** | **Mean systolic I.O. pressure**  **(mmHg)** | **Mean diastolic I.A. pressure (mmHg)** | **Mean diastolic I.O. pressure**  **(mmHg)** | **Pearson’s r**  **systolic** | **Pearson’s r**  **diastolic** |
| --- | --- | --- | --- | --- | --- | --- | --- | --- |
| Normotension (n=6) | 68.6 (22.1) | 29.1 (4.6) | 95.5 | 37.3 | 52.4 | 27.8 | 0.88 | 0.66 |
| Hypotension (n=2) | 54.8 (17.7) | 30.3 (4.1) | 82.4 | 38.3 | 46.3 | 26.8 | 0.94 | 0.52 |
| Pacing 140bpm (n=2) | 60.7(18.2) | 31.8(4.1) | 85 | 37 | 50.6 | 29.6 | 0.91 | 0.58 |
| Ventricular tachycardia (n=1) | 61.5(18.4) | 37(4.8) | 86.6 | 44 | 54.1 | 35.5 | 0.53 | 0.935 |
| Breath hold (n=2) | 80.6 (22.3) | 36.2(2.6) | 103.8 | 39.4 | 68 | 34.1 | 0.97 | 0.87 |
| Epinephrine (200mg) (n=1) | 93.5(35.6) | 29.3(3.8) | 124 | 34.5 | 71.9 | 27.8 | -0.384 | -0.202 |
